# Redox-Active Anthraquinone-1-Sulfonic Acid Sodium Salt-Loaded Polyaniline for Dual-Functional Electrochromic Supercapacitors

**DOI:** 10.3390/gels11080568

**Published:** 2025-07-23

**Authors:** Yi Wang, Enkai Lin, Ze Wang, Tong Feng, An Xie

**Affiliations:** 1 Key Laboratory of Functional Materials and Applications of Fujian Province, School of Materials Science and Engineering, Xiamen University of Technology, Xiamen 361024, China; 19859769527@163.com (E.L.); 2322161045@stu.xmut.edu.cn (Z.W.); anxie@xmut.edu.cn (A.X.); 2 National Key Laboratory of Electronic Thin Films and Integrated Devices, National Engineering Research, University of Electronic Science and Technology of China, Chengdu 610054, China; 3 School of Mechanical Electrical and Information Engineering, Xiamen Institute of Technology, Xiamen 361021, China

**Keywords:** electrochromic, supercapacitor, polyaniline, anthraquinone-1-sulfonic acid sodium salt, redox gel electrolyte

## Abstract

Electrochromic (EC) devices are gaining increasing attention for next-generation smart windows and low-power displays due to their reversible color modulation, low operating voltage, and flexible form factors. Recently, electrochromic energy storage devices (EESDs) have emerged as a promising class of multifunctional systems, enabling simultaneous energy storage and real-time visual monitoring. In this study, we report a flexible dual-functional EESD constructed using polyaniline (PANI) films doped with anthraquinone-1-sulfonic acid sodium salt (AQS), coupled with a redox-active PVA-based gel electrolyte also incorporating AQS. The incorporation of AQS into both the polymer matrix and the gel electrolyte introduces synergistic redox activity, facilitating bidirectional Faradaic reactions at the film–electrolyte interface and within the bulk gel phase. The resulting vertically aligned PANI-AQS nanoneedle films provide high surface area and efficient ion pathways, while the AQS-doped gel electrolyte contributes to enhanced ionic conductivity and electrochemical stability. The device exhibits rapid and reversible color switching from light green to deep black (within 2 s), along with a high areal capacitance of 194.2 mF·cm^−2^ at 1 mA·cm^−2^ and 72.1% capacitance retention over 5000 cycles—representing a 31.5% improvement over undoped systems. These results highlight the critical role of redox-functionalized gel electrolytes in enhancing both the energy storage and optical performance of EESDs, offering a scalable strategy for multifunctional, gel-based electrochemical systems in wearable and smart electronics.

## 1. Introduction

Electrochromism, a pivotal branch of chromogenic technologies, offers tunable coloration, low operating voltages, and facile control, conferring substantial commercial potential in smart-window, display, and related markets [1,2,3,4]. With the continuous advancement of technology, traditional rigid electrochromic devices limited to single-function color modulation are increasingly insufficient to meet the evolving demands of next-generation applications. Consequently, flexible electrochromic (EC) devices with integrated multifunctional capabilities have emerged as a prominent focus of current research [5,6]. Owing to the structural resemblance between EC cells and supercapacitors, multifunctional EC–supercapacitor architectures—capable of visually indicating residual charge by color change—have attracted considerable attention [7,8].

Polyaniline (PANI), a well-known conducting polymer, has emerged as a promising candidate for both electrochromic and energy-storage applications owing to its low cost, ease of synthesis, reversible redox transitions, tunable doping levels, high environmental stability, and rich color states resulting from its oxidation-state-dependent optical properties [9,10]. Recently, there has been increasing interest in electrochromic energy storage devices (EESDs), which integrate optical modulation and energy storage into a single platform. Several reports have explored PANI-based EESDs. For instance, Tian et al. [11] prepared a coral-like PANI/MnO_2_ composite film via one-step electrochemical co-deposition and constructed a complementary EESD with WO_3_ as the counter electrode, enabling real-time monitoring of device state through color change. Sui et al. [12] synthesized PANI/MoO_3−x_ core–shell composites showing high capacitance, good electrochromic performance, and flexibility for solid-state supercapacitors. Ouyang et al. [13] prepared CuPc-PANI composite films via in situ electrochemical polymerization, which showed enhanced electrochemical and electrochromic performance compared to pure PANI. Furthermore, Wu et al. [14] prepared a hydrogen-bonded graphene/PANI composite with high capacitance (598 F g^−1^) and fast, reversible color change. Despite recent advances, the development of high-performance electrochromic energy storage devices still faces significant challenges, particularly in terms of long-term stability and energy storage capacity. In our previous work, pyrene tetrasulfonic acid tetrasodium salt (PTSA) was incorporated into the PANI matrix to improve its mechanical integrity and electrochemical cycling stability, benefiting from PTSA’s rigid aromatic core and multiple sulfonate groups that provide strong electrostatic interactions with the polymer backbone [15]. However, PTSA is electrochemically inert and thus does not contribute to the overall charge-storage capacity of the system.

Generally, during the redox processes of PANI, dopant counterions are reversibly inserted and extracted to maintain charge neutrality, playing a critical role in driving the Faradaic transitions of the polymer electrode. Common ionic dopants—such as perchlorate (ClO_4_^−^), sulfate (SO_4_^2−^), and chloride (Cl^−^)—are spontaneously incorporated into the polymer network during electrochemical polymerization via electrostatic interactions. However, these small anionic species are redox-inactive and therefore do not directly contribute to the energy storage capability of EESDs. To overcome this limitation, introducing redox-active dopants has emerged as an effective strategy to enhance both the electrochromic and capacitive performance of PANI. Such dopants not only maintain electronic conductivity by facilitating charge compensation but also participate in reversible Faradaic reactions, thereby providing additional pseudocapacitance and enabling synergistic enhancement of dual functionalities in EESDs.

In this study, we employ anthraquinone-1-sulfonic acid sodium salt (AQS), a redox-active dopant, as both a film modifier and an electrolyte additive. Quinone and its derivatives are considered among the most attractive pseudocapacitive materials due to their low cost, highly reversible redox activity, fast electron transfer kinetics, and environmental sustainability. AQS, as a water-soluble anthraquinone derivative, is particularly suitable for use in EESDs [16]. The sulfonate groups of AQS facilitate strong electrostatic interactions with the PANI backbone, enhancing the structural integrity and cycling stability of the resulting composite during repeated charge–discharge processes. Moreover, AQS offers substantial theoretical specific capacitance through a reversible two-electron redox reaction between the carbonyl groups and protons (H^+^), contributing additional Faradaic charge storage [17]. By optimizing the molar ratio of AQS to aniline, we obtained vertically aligned nanoneedle PANI-AQS films on flexible Au/Nylon-66 substrates, offering high surface area and abundant ion pathways. AQS doping markedly enhances the film’s capacitance and cycling durability without compromising its optical modulation. The assembled thin-film device exhibits pronounced color switching from light green to deep black with applied potential, while the AQS-based redox electrolyte further improves electrochemical performance. Together, these results justify the rational selection of AQS as a multifunctional dopant, highlighting its dual role in enhancing both electrochromic contrast and energy storage capability.

## 2. Results and Discussion

### 2.1. Optimization, Preparation, and Characterization of PANI-AQS Films

**Figure 1:**
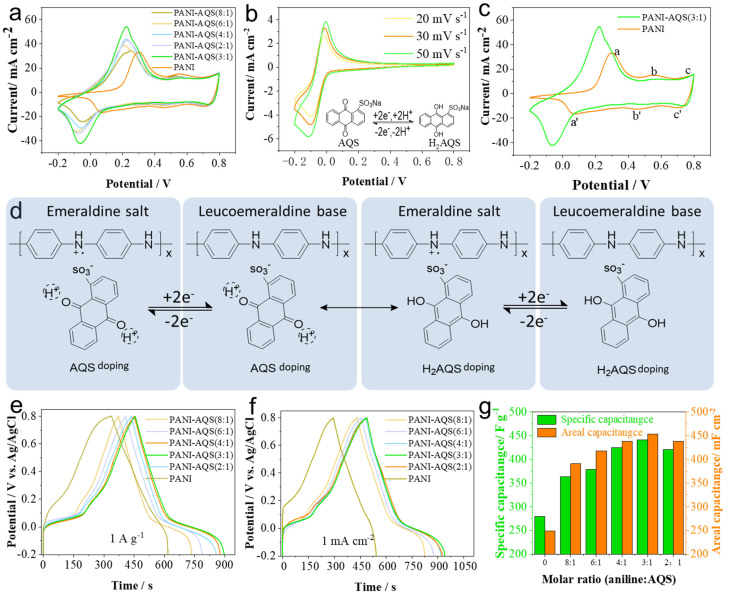
Electrochemical performance of different film samples in a three-electrode system and the interaction mechanism between AQS and PANI chains. (**a**) Cyclic voltammetry curves of different film electrodes at a scan rate of 50 mV s^−1^; (**b**) cyclic voltammetry curve of AQS molecules; (**c**) cyclic voltammetry curves comparison between PANI-AQS(3:1) and pure PANI films; (**d**) schematic illustration of the interaction between PANI chains and AQS; (**e**) galvanostatic charge–discharge curves of film electrodes at a current density of 1 A g^−1^; (**f**) galvanostatic charge–discharge curves of film electrodes at a current density of 1 mA cm^−2^; (**g**) specific capacitance and areal capacitance statistics of films with different molar ratios.

Electrochemical tests were performed on a series of PANI-based films, and their cyclic voltammetry (CV) curves are shown in Figure 1a. In this study, samples labeled as PANI-AQS(x:y) refer to films electrochemically deposited using precursor solutions with a molar ratio of aniline monomer to AQS of x:y. Compared to the pristine PANI film electrode, the AQS-modified film electrodes exhibited significantly enhanced electrochemical performance. Among them, the PANI-AQS(3:1) film showed the highest peak current and the largest CV enclosed area, indicating the highest specific capacitance. Moreover, the CV curves reveal a notable difference in the redox peaks within the potential range of −0.2 to 0.3 V for PANI-AQS(3:1) compared to the pure PANI film, which is attributed to the effect of AQS modification. Since AQS molecules possess redox activity, their redox potentials were further investigated by CV using a three-electrode system with 1 mol L^−1^ H_2_SO_4_ as the supporting electrolyte and 0.01 mol L^−1^ AQS added. Two platinum electrodes served as working and counter electrodes, respectively, and Ag/AgCl was the reference electrode. The CV curve of AQS, shown in Figure 1b, exhibits a pair of well-defined redox peaks between −0.2 and 0.1 V. With increasing scan rates, these peaks shift positively. The inset in Figure 1b illustrates the molecular structure transformation during the redox process [16,18].

To further analyze the distinct CV behavior of the optimized PANI-AQS(3:1) film compared to pure PANI, their CV curves are presented in Figure 1c. The PANI film exhibits three pairs of characteristic redox peaks (a/a′ to c/c′). The first pair (a/a′) corresponds to the redox transition between the leucoemeraldine base (LB, fully reduced form) and emeraldine salt (ES, partially oxidized form). The second pair (b/b′) is attributed to the further oxidation within the emeraldine state. The third pair (c/c′) represents the transition from the emeraldine to the pernigraniline state, involving the formation of quinoneimine structures [19,20]. Combining the CV characteristics of AQS and pure PANI, it is evident that in the potential range of −0.2 to 0.3 V, PANI-AQS(3:1) undergoes both AQS redox reactions and the LB-ES interconversion of PANI. This molecular transformation during the redox process is schematically illustrated in Figure 1d, where AQS contributes to charge storage via a Faradaic pseudocapacitive mechanism within this voltage window. Overall, the comparison of CV curves demonstrates that the introduction of AQS effectively enhances the pseudocapacitive behavior of the composite PANI-AQS(3:1) film.

The galvanostatic charge–discharge (GCD) performance of the film electrodes was evaluated in a three-electrode system at current densities of 1 A g^−1^ and 1 mA cm^−2^, as shown in Figure 1e,f. The combined CV and GCD results indicate that the film with an aniline-to-AQS molar ratio of 3:1 exhibits the best electrochemical energy storage performance, achieving specific capacitances of 441.2 F g^−1^ at 1 A g^−1^ and 453 mF cm^−2^ at 1 mA cm^−2^. The histogram of areal and gravimetric capacitances for films with different doping ratios is presented in Figure 1g. Compared to the pristine PANI film (279.7 F g^−1^ at 1 A g^−1^ and 248.6 mF cm^−2^ at 1 mA cm^−2^), the AQS-modified films show significant performance enhancement.

**Figure 2:**
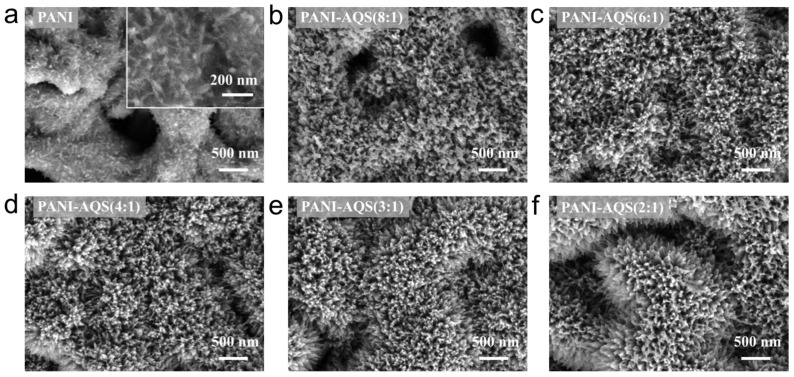
SEM characterization of the films: (**a**) PANI film; (**b**) surface morphology of PANI-AQS (8:1) film; (**c**) PANI-AQS (6:1) film; (**d**) PANI-AQS (4:1) film; (**e**) PANI-AQS(3:1) film; (**f**) PANI-AQS (2:1) film.

Surface morphology analysis of films with varying AQS content is shown in Figure 2a–f. The pristine PANI sample exhibits a fine nanowire morphology (Figure 2a). In contrast, AQS doping induces a vertically aligned nanoneedle structure, which becomes more distinct and elongated as the AQS molar ratio increases. The PANI-AQS(3:1) sample (Figure 2e) maintains abundant mesopores between densely packed nanoneedles, correlating with its superior electrochemical performance observed in the three-electrode tests. Compared to pure PANI films, the nanoneedle morphology induced by AQS doping greatly increases the specific surface area of the films, facilitating improved electrolyte–electrode contact and providing more electrochemical active sites, thereby enhancing the overall electrochemical performance [21].

Material composition analysis and characterization were performed on the prepared PANI films and the optimized PANI-AQS(3:1) samples. Energy-dispersive X-Ray spectroscopy (EDS) mapping of the film surfaces was conducted, as shown in Figure S1. The elemental distribution maps reveal a uniform dispersion of all detected elements across the surface, indicating excellent film homogeneity. X-Ray photoelectron spectroscopy (XPS) characterization of the PANI-AQS(3:1) film surface is presented in Figure S2. The high-resolution O 1s spectrum clearly shows a distinct C=O peak after AQS modification, confirming the successful incorporation of AQS into the polymer matrix via electrostatic interactions [22]. Further analysis was conducted on pure PANI films oxidized in 0.01 M AQS-containing H_2_SO_4_ electrolyte. The O 1s spectrum of these oxidized samples closely resembles that of the PANI-AQS(3:1) film, suggesting the occurrence of anion doping exchange during the oxidation process. Additionally, weak O 1s peaks associated with perchlorate ions were detected in both samples, indicating that perchlorate anions also participate in anion doping of the PANI chains during film preparation [23].

### 2.2. Electrochemical Performance Study of PANI-AQS Films

**Figure 3:**
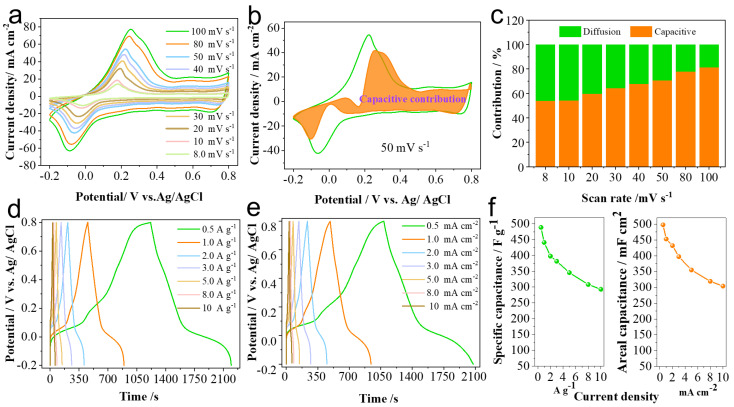
Electrochemical performance of PANI-AQS(3:1) film electrode in a three-electrode system. (**a**) Cyclic voltammetry curves of the film electrode at various scan rates; (**b**) capacitive current contribution (orange shaded area) and total current from cyclic voltammetry curve at 50 mV s^−1^; (**c**) histogram of capacitive contribution percentages at different scan rates; (**d**) galvanostatic charge–discharge curves used to calculate mass-specific capacitance; (**e**) galvanostatic charge–discharge curves used to determine areal capacitance; (**f**) statistical summary of capacitance values for different film samples.

The electrochemical performance of the PANI-AQS(3:1) film electrode at the optimized ratio was systematically investigated. Cyclic voltammetry (CV) curves of the PANI-AQS(3:1) electrode were recorded at various scan rates ranging from 8 to 100 mV s^−1^, as shown in Figure 3a. The CV curves maintain their shape well even at high scan rates, indicating favorable electrochemical kinetics and excellent rate capability of the film. To further analyze the energy storage mechanism of the film electrode, the capacitive contribution was calculated using the Bruce–Dunn method [24]. The fitted CV curve at a scan rate of 50 mV s^−1^ is shown in Figure 3b, where the capacitive-controlled contribution (highlighted in orange) accounts for 70.5% of the total capacitance. Figure 3c illustrates the capacitive contribution percentages at scan rates of 8, 10, 20, 30, 40, 50, 80, and 100 mV s^−1^, which are 53.8%, 54.1%, 59.6%, 64.0%, 67.6%, 70.5%, 77.6%, and 81.2%, respectively. These results demonstrate that surface-controlled pseudocapacitance dominates the charge storage process of the film material. Galvanostatic charge–discharge (GCD) curves of the PANI-AQS(3:1) electrode were recorded in 1 mol L^−1^ H_2_SO_4_ electrolyte at different current densities, as shown in Figure 3d,e. The PANI-AQS electrode delivers specific capacitances of 489 F g^−1^ and 441.2 F g^−1^ at current densities of 0.5 A g^−1^ and 1 A g^−1^, respectively. Areal capacitances of 498 mF cm^−2^ and 453 mF cm^−2^ were obtained at current densities of 0.5 mA cm^−2^ and 1 mA cm^−2^, respectively. The capacitance values at various current densities are summarized in Figure 3f.

### 2.3. Effect of AQS Electrolyte Additive on the Electrochemical Performance of Films

Given the inherent redox activity of AQS, its role as an electrolyte additive in enhancing the electrochemical performance of the film electrodes was further investigated. The electrolyte containing the additive was prepared by dissolving AQS powder in 1 mol·L^−1^ H_2_SO_4_, with the final concentration of AQS controlled at 0.01 mol·L^−1^. For clarity, the notation used in the Figures is as follows: PANI—[H_2_SO_4_] and PANI-AQS(3:1)—[H_2_SO_4_] represent PANI and PANI-AQS(3:1) films tested in conventional 1 mol L^−1^ H_2_SO_4_ electrolyte, respectively; PANI—[H_2_SO_4_+AQS] and PANI-AQS(3:1)—[H_2_SO_4_+AQS] denote the corresponding films tested in H_2_SO_4_ electrolyte containing the AQS additive.

**Figure 4:**
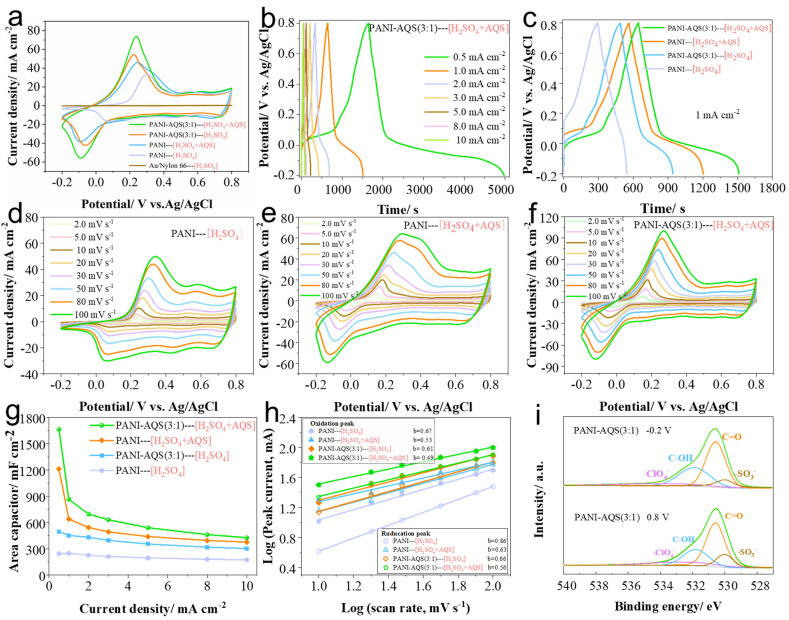
Electrochemical performance of films in a three-electrode system. (**a**) Cyclic voltammetry curves; (**b**) galvanostatic charge–discharge curves of the PANI-AQS(3:1) film electrode; (**c**) galvanostatic charge–discharge profiles of PANI and PANI-AQS(3:1) films measured in various electrolyte systems; (**d**–**f**) cyclic voltammetry curves of films at varying scan rates; (**g**) areal capacitance statistics of film electrodes at different current densities; (**h**) logarithmic plots of peak current (log i) versus scan rate (log v) for different film electrodes; (**i**) X-Ray photoelectron spectroscopy (XPS) spectra.

Figure 4a presents the CV curves of different film samples (Au/Nylon 66 electrode, PANI, and PANI-AQS(3:1)) measured in a three-electrode system with different electrolytes. The flexible Au/Nylon 66 electrode without active material shows negligible CV area in H_2_SO_4_ electrolyte, indicating minimal electrochemical capacitance contribution. Comparing the CV curves of PANI and PANI-AQS(3:1) films in H_2_SO_4_ electrolyte with those in H_2_SO_4_+AQS additive electrolyte reveals a pronounced increase in enclosed CV area upon addition of AQS. Notably, the PANI-AQS(3:1)—[H_2_SO_4_+AQS] sample exhibits the largest CV area, demonstrating that AQS doping in the electrolyte further enhances the charge storage capacity. GCD measurements of the PANI-AQS(3:1) film in the electrolyte containing the additive are shown in Figure 4b. The discharge capacity of the film electrode in the electrolyte with AQS additive significantly exceeds that in the pure H_2_SO_4_ electrolyte. Under current densities of 0.5 mA cm^−2^ and 1 mA cm^−2^, the PANI-AQS(3:1) electrode delivers areal capacitances of 1666.5 mF cm^−2^ and 865 mF cm^−2^, respectively. Figure 4c compares GCD curves of different samples at 1 mA cm^−2^, where the extended discharge time confirms the substantial enhancement of capacitance due to AQS doping in the electrolyte.

CV curves at various scan rates for different samples are displayed in Figure 4d–f. The PANI-AQS(3:1)—[H_2_SO_4_+AQS] sample shows higher peak currents and larger enclosed CV areas than both PANI—[H_2_SO_4_] and PANI—[H_2_SO_4_+AQS] samples, consistent with GCD results. Additionally, as the scan rate increases, the CV curves exhibit a uniform increase in current while maintaining good shape, indicating favorable electrochemical kinetics. Figure 4g summarizes the areal capacitance values of different samples at various current densities. It is evident that regardless of whether AQS is incorporated into the PANI matrix via anion doping during electrochemical deposition or added directly as a redox-active additive in the electrolyte, the electrochemical energy storage performance of the PANI-based films is significantly improved.

Further analysis was conducted on the energy storage kinetics of PANI-based films in different electrolytes. The relationship between the peak current (i) of the most prominent redox couple in the CV curves and the scan rate (v) was examined by plotting log(i) versus log(v) [25], as shown in Figure 4h. The fitting results indicate that for both PANI and PANI-AQS(3:1) films tested in electrolytes containing AQS, the calculated b-values at the most distinct redox peaks are lower than those obtained without AQS additives. This suggests that the introduced AQS, under an applied electric field, utilizes its anthraquinone functional groups to undergo Faradaic reactions at the electrode–electrolyte interface, thereby enhancing ion storage from the electrolyte and consequently increasing the capacitance of the film electrodes [26]. Moreover, XPS analysis was performed on PANI-AQS(3:1) films charged to 0.8 V and discharged to −0.2 V in 1 mol L^−1^ H_2_SO_4_ electrolyte, focusing on the O 1s spectra shown in Figure 4i. At −0.2 V, the relative proportion of C–OH species is significantly higher than that of C=O, indicating that during the discharge process to −0.2 V, a portion of C=O groups undergo proton-coupled reduction to form C–OH. Conversely, at 0.8 V, the C=O content is higher than at −0.2 V. The reversible conversion of carbonyl groups in AQS endows the AQS-doped PANI films with enhanced specific capacitance. Additionally, the presence of AQS in the electrolyte facilitates Faradaic reactions at the electrode interface, further improving the capacitance of the film electrodes [17].

The cycling stability of the film samples was evaluated in a three-electrode system using different electrolytes. Both PANI and PANI-AQS(3:1) films were tested in 1 mol L^−1^ H_2_SO_4_ and in 0.01 mol L^−1^ AQS+1 mol L^−1^ H_2_SO_4_ electrolytes. As shown in Figure S3, the results demonstrate that the stability of the PANI film in 1 mol L^−1^ sulfuric acid is relatively poor, retaining only 46% of its initial capacitance after 1000 cycles. However, with the addition of AQS to the electrolyte, the cycling stability improved significantly, with a retention rate of 61.8% after 1000 cycles. In comparison, the PANI-AQS(3:1) samples exhibited markedly better stability than the pure PANI films. Specifically, the PANI-AQS(3:1) films maintained 69% and 76.4% of their initial capacitance after 1000 cycles in 1 mol L^−1^ H_2_SO_4_ and 0.01 mol L^−1^ AQS+1 mol L^−1^ H_2_SO_4_ electrolytes, respectively. These results indicate that the incorporation of AQS effectively enhances the electrochemical cycling stability of PANI films.

### 2.4. Electrochromic Performance of PANI-AQS Films

**Figure 5:**
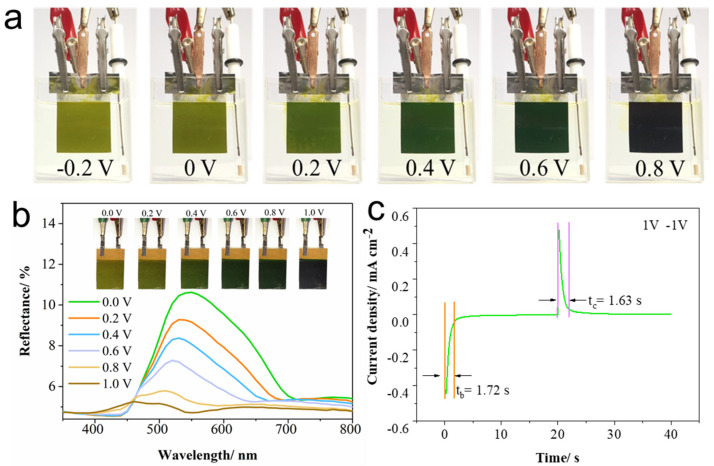
(**a**) Optical photographs shown the color change of PANI-AQS(3:1) film in AQS-doped electrolyte under a three-electrode system; (**b**) reflectance spectra of the film device at different discharge voltages; (**c**) step potential response testing at +1 V and −1 V.

The electrochromic properties of the films were investigated. Optical images of the color changes of the PANI-AQS(3:1) film in a three-electrode system with 0.01 mol L^−1^ AQS+1 mol L^−1^ H_2_SO_4_ electrolyte are shown in Figure 5a. The film exhibited excellent visible-light electrochromic behavior, transitioning from light green to dark green and finally to deep black as the applied voltage increased. This pronounced electrochromic response can serve as a visual indicator of the film’s charge state, suggesting promising potential for the development of high-performance electrochromic energy storage devices.

Further, the response times of the PANI-AQS(3:1) film in a three-electrode system were evaluated. As shown in Figure S4, in situ current response measurements were conducted under step potentials of −0.2 V and 0.8 V. The response speeds were calculated from the current–time curves, yielding coloration and bleaching response times of 1.35 s and 1.06 s, respectively, demonstrating the film’s rapid electrochromic switching behavior.

### 2.5. Performance Study of Dual-Functional Electrochromic-Supercapacitor Film Devices

Based on the systematic electrochemical investigations under three-electrode configurations, it is evident that the incorporation of AQS—whether as an anionic dopant within the PANI polymer chains during electropolymerization or as an additive in the electrolyte solution—significantly enhances the electrochemical properties of the films. Building upon these findings, symmetric dual-functional electrochromic-supercapacitor devices were fabricated using the optimized PANI-AQS(3:1) films. The devices employed an AQS-doped gel electrolyte, specifically 0.01 mol L^−1^ AQS incorporated into a 1 mol L^−1^ PVA/H_2_SO_4_ matrix, thus integrating electrochromic and energy storage functionalities in a single system. The electrochromic behavior of the device was evaluated by measuring the reflectance spectra within the 350–800 nm wavelength range under varying discharge voltages from 0 to 1 V (Figure 5b). At 0 V, the device exhibited high reflectance attributable to the high transmittance state of PANI and the reflective nature of the underlying Au electrode. As the applied voltage increased, a gradual decrease in reflectance was observed, accompanied by a continuous and reversible color change from light green to dark green and ultimately to near-black. This tunable optical modulation confirms the device’s capability for colorimetric indication of its charge state. Dynamic response tests were conducted using step voltages of +1 V and −1 V to probe the device’s electrochromic switching kinetics (Figure 5c). The calculated coloring and bleaching times were 1.72 s and 1.63 s, respectively, demonstrating rapid and stable color switching performance suitable for real-time applications.

**Figure 6:**
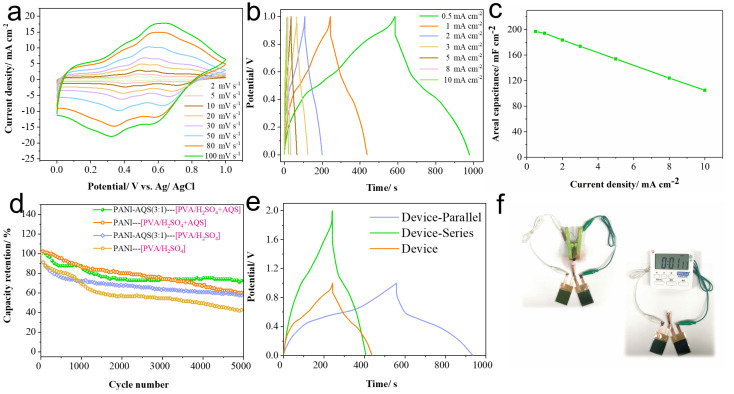
Electrochemical performance testing and demonstration of the film devices. (**a**) Cyclic voltammetry curves of the device; (**b**) galvanostatic charge–discharge curves of the device; (**c**) areal capacitance values at various current densities; (**d**) cycling stability test of the device; (**e**) galvanostatic charge–discharge testing of devices connected in series and parallel configurations; (**f**) demonstration of the device powering different small electronic appliances.

Electrochemical energy storage properties were further characterized by CV at different scan rates (Figure 6a). The CV curves showed a proportional increase in enclosed area with scan rate while maintaining consistent shape, indicative of excellent capacitive behavior [27]. GCD measurements (Figure 6b) revealed areal capacitances of 197 mF cm^−2^ and 194.2 mF cm^−2^ at current densities of 0.5 mA cm^−2^ and 1 mA cm^−2^, respectively. The device retained over 53.3% of its capacitance when the current density was increased by a factor of 20 (Figure 6c), highlighting its outstanding rate capability. Long-term cycling stability tests (Figure 6d) demonstrated that in the redox-active AQS-containing electrolyte, the PANI-AQS(3:1) devices retained 72.1% of their initial capacitance after 5000 cycles, outperforming the undoped PANI devices, which maintained 60%. In contrast, in the absence of redox additives, capacitance retention dropped to 57.6% and 40.6% for the doped and undoped devices, respectively. These results underscore the beneficial role of AQS doping or electrolyte modification in enhancing cycling durability.

To evaluate practical scalability, devices were subjected to series and parallel configurations with subsequent GCD testing (Figure 6e). Series-connected devices exhibited extended operating voltage windows with only slight reductions in discharge time, while parallel connections nearly doubled the overall capacitance compared to single devices, confirming effective performance scaling. Finally, the applicability of the dual-functional device was demonstrated by powering small-scale electronic components (Figure 6f). The device delivered stable energy output and simultaneously enabled real-time electrochromic visualization of the charge state, highlighting its potential integration with wearable and smart electronic systems for energy storage with visual feedback.

## 3. Conclusions

To enhance the electrochemical performance of PANI-based electrochromic-supercapacitor bifunctional film devices, various dopants or modifying additives are typically employed. However, these conventional additives often lack electrochemical activity, thereby limiting the extent of performance improvement. In this work, AQS as a redox-active molecule is used both as a structural dopant in the PANI film and as a redox additive in the electrolyte solution. This strategy enables the fabrication of AQS-modified PANI-AQS films with vertically aligned nanoneedle-like morphologies. The presence of AQS facilitates Faradaic reactions either within the film matrix or at the electrode–electrolyte interface, enhancing the film’s electrochemical performance via a redox pseudocapacitive mechanism. The nanoneedle morphology of the PANI-AQS films provides a high specific surface area and a porous structure, which promote efficient ion diffusion and abundant electroactive sites. During the reversible transition between the reduced and conductive states of PANI, AQS anions serve as dopants that intercalate into the polymer chains, further boosting the charge storage capability due to their intrinsic redox activity. The resulting PANI-AQS film device exhibits excellent electrochromic performance. It shows a continuous color transition from light green to dark green to deep black, which enables real-time visual estimation of the charge state. The electrochromic switching response time is less than 2 s. Furthermore, AQS can be incorporated into the gel electrolyte to form a redox-active electrolyte. This significantly improves the device’s overall electrochemical performance. When tested in a redox electrolyte doped with AQS, the PANI-AQS film device retained 72.1% of its initial capacitance after 5000 cycles—an improvement of 31.5% compared to a PANI film in a conventional electrolyte. Additionally, the device delivered a high areal capacitance of 194.2 mF cm^−2^ at a current density of 1 mA cm^−2^.

## 4. Materials and Methods

### 4.1. Materials

Aniline and anthraquinone-1-sulfonic acid sodium salt (AQS) were purchased from Aladdin Reagents Co., Ltd. (Shanghai, China) The poly(vinyl alcohol) (PVA) used in this study was purchased from Alfa Aesar (Ward Hill, MA, USA) and is specified as medium molecular weight with a hydrolysis degree of 98–99%. Perchloric acid (HClO_4_) and sulfuric acid (H_2_SO_4_) were obtained from Kelong Chemical Reagents Co., Ltd. (Chengdu, China) Au/Nylon 66 flexible electrodes were prepared following the procedure previously reported by our group [28,29].

### 4.2. Preparation of Electrochemical Deposition Solutions

An aqueous solution of perchloric acid (1 mol L^−1^) was first prepared. Subsequently, aniline monomer was added dropwise to the solution to achieve a final concentration of 0.03 mol L^−1^. This solution was used for the electrochemical deposition of PANI films without AQS doping. To prepare PANI-AQS(x:y) composite films, AQS powder was further introduced into the aforementioned solution, with the molar ratios of aniline to AQS precisely adjusted to 8:1, 6:1, 4:1, 3:1, and 2:1, respectively. The resulting films were designated as PANI-AQS(x:y), where x:y denotes the molar ratio between aniline and AQS.

### 4.3. Fabrication of PANI-AQS(x:y) Electrodes and Film Device

The fabrication of PANI-AQS(x:y) electrodes followed a protocol adapted from previous reports [15]. Electrochemical deposition was performed using an Au/Nylon 66 substrate as the working electrode, a platinum sheet as the counter electrode, and an Ag/AgCl electrode as the reference. The pre-prepared deposition solution was employed throughout. First, cyclic voltammetry (CV) was carried out between 0 and 1 V at 50 mV s^−1^ for five cycles. Upon completion, the electrode was removed and rinsed thoroughly with deionized water. Next, galvanostatic deposition was conducted at a current density of 0.25 mA cm^−2^ for 8000 s. Finally, the electrode was rinsed again with deionized water and air-dried.

The assembly procedure of the PANI film device is as follows: First, the gel electrolyte is uniformly applied to both sides of the electrode films and allowed to fully penetrate into the Nylon 66 filter membrane. Then, the two electrodes are assembled into a sandwich structure and encapsulated with a transparent PE film. During assembly, both PANI films face outward, resulting in a reflective bifunctional film device.

### 4.4. Preparation of Gel Electrolytes

A total of 1 g of concentrated H_2_SO_4_ was slowly added dropwise into 10 mL of deionized water under stirring. Then, 1 g of PVA powder was added to the solution, which was heated and stirred at 85 °C until it formed a transparent gel, resulting in an H_2_SO_4_/PVA gel electrolyte. For the preparation of the AQS-doped electrolyte, the same procedure was followed, except that AQS powder was further added to the above solution to a final AQS concentration of 0.01 mol L^−1^. The solution mixed with AQS was further heated and stirred at 85 °C until a clear, light-yellow gel electrolyte was obtained.

### 4.5. Characterization

The surface morphology of the Au/Nylon 66 substrates and PANI-based electrode films was investigated using a field emission scanning electron microscope (FE-SEM, GeminiSEM 300; Carl Zeiss, Oberkochen, Germany), equipped with an energy-dispersive X-Ray spectroscopy (EDS) system (Smartedx; Hitachi, Tokyo, Japan). The elemental composition of the samples was analyzed by X-Ray photoelectron spectroscopy (XPS, Kratos XSAM800; Kratos Analytical Ltd., Manchester, UK). The optical reflectance spectra of the electrochromic films were recorded using a PerkinElmer Frontier FT-IR spectrometer (Frontier; PerkinElmer Inc., Waltham, MA, USA) operating in the UV–Vis–NIR range. Electrochemical measurements were performed using a CHI660E electrochemical workstation (CH Instruments, Shanghai, China). In the three-electrode system, the prepared PANI-based film served as the working electrode, Ag/AgCl as the reference electrode, and a platinum electrode as the counter electrode. The supporting electrolyte was either 1 mol L^−1^ H_2_SO_4_ solution or an AQS-doped electrolyte solution. For device testing, the films were encapsulated with the prepared gel electrolyte, and the relevant testing conditions have been clearly described.

## Data Availability

The original contributions presented in this study are included in the article. Further inquiries can be directed to the corresponding author.

## Supplementary Materials

The following supporting information can be downloaded at https://www.mdpi.com/article/10.3390/gels11080568/s1: Figure S1. EDS mapping characterization of the PANI-AQS(3:1) film surface. Figure S2. XPS characterization and analysis of the PANI-AQS(3:1) film surface. Figure S3. Cycling performance study of the film in a three-electrode system. Figure S4. Response time of the PANI-AQS(3:1) film in a three-electrode system.
