# Supplementary material for: Redox-Active Anthraquinone-1-Sulfonic Acid Sodium Salt-Loaded Polyaniline for Dual-Functional Electrochromic Supercapacitors
Source: Gels. 2025 Jul 23;11(8):568. doi: 10.3390/gels11080568 (PMC12385569; doi:10.3390/gels11080568)
Supplement: Supplementary file 1 [file gels-11-00568-s001.zip › gels-3754747-supplementary.pdf]

Supporting Information

Redox-Active Anthraquinone-1-Sulfonic Acid Sodium Salt-Loaded Polyaniline for Dual-Functional Electrochromic Supercapacitors

Yi Wang[1,2*], Enkai Lin[1], Ze Wang[1], Tong Feng[3*], An Xie[1]

[1]*Key Laboratory of Functional Materials and Applications of Fujian Province, School of Materials Science and Engineering, Xiamen University of Technology, Xiamen 361024, PR China.*

[2]*National Key Laboratory of Electronic Thin Films and Integrated Devices, National Engineering Research, University of Electronic Science and Technology of China, Chengdu 610054, China.*

[3]*School of Mechanical Electrical and Information Engineering, Xiamen Institute of Technology, Xiamen, 361021, PR China.*

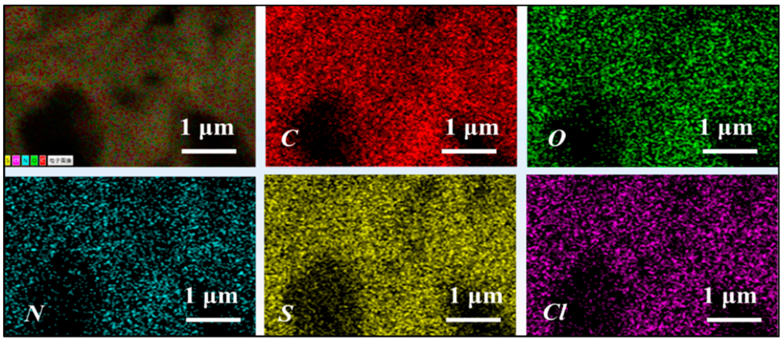

Figure S1. EDS mapping characterization of the PANI-AQS (3:1) film surface.

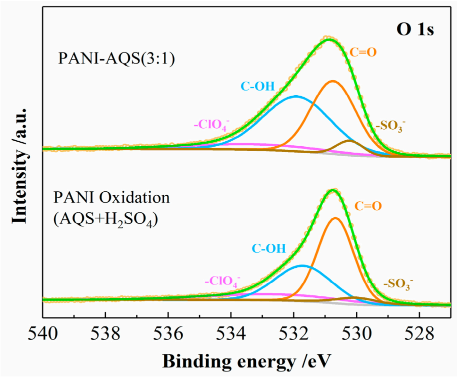

Figure S2. XPS characterization and analysis of the PANI-AQS (3:1) film surface.

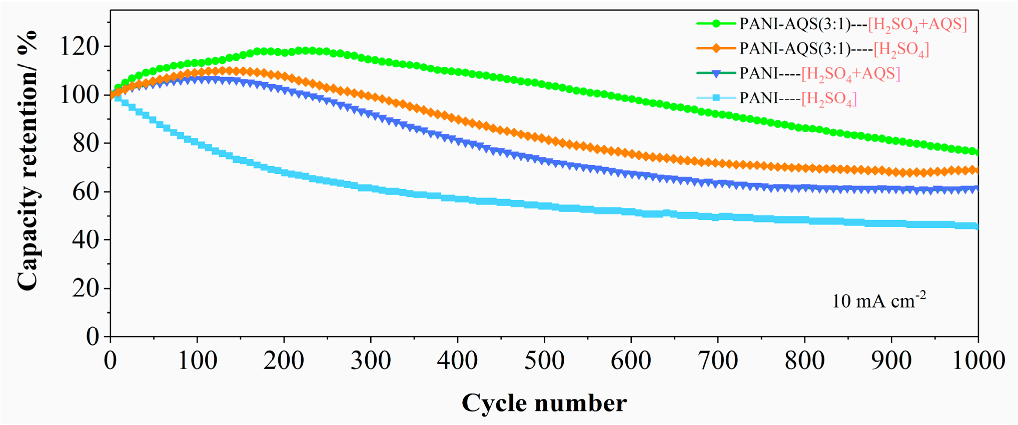

Figure S3. Cycling performance study of the film in a three-electrode system.

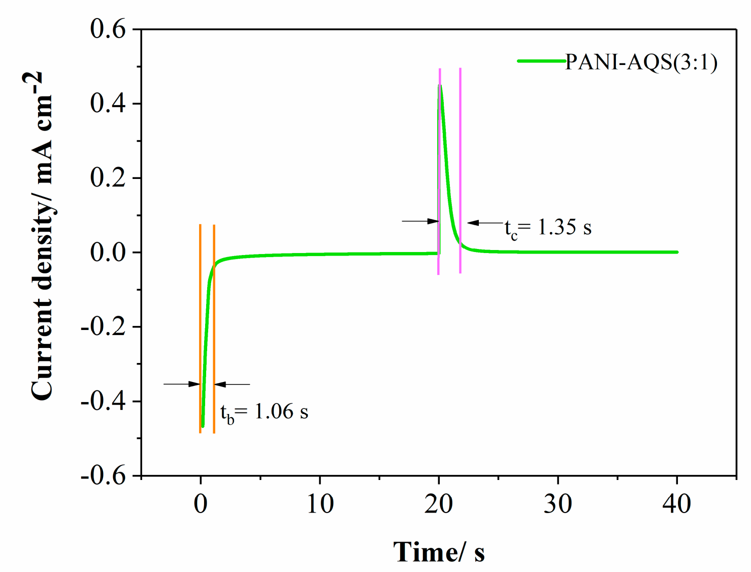

Figure S4. Response time of the PANI-AQS (3:1) film in a three-electrode system.
